# Autonomously revealing hidden local structures in supercooled liquids

**DOI:** 10.1038/s41467-020-19286-8

**Published:** 2020-10-30

**Authors:** Emanuele Boattini, Susana Marín-Aguilar, Saheli Mitra, Giuseppe Foffi, Frank Smallenburg, Laura Filion

**Affiliations:** 1 Soft Condensed Matter, Debye Institute of Nanomaterials Science, Utrecht University, Utrecht, Netherlands; 2 Université Paris-Saclay, CNRS, Laboratoire de Physique des Solides, 91405 Orsay, France

**Keywords:** Structure of solids and liquids, Statistical physics

## Abstract

Few questions in condensed matter science have proven as difficult to unravel as the interplay between structure and dynamics in supercooled liquids. To explore this link, much research has been devoted to pinpointing local structures and order parameters that correlate strongly with dynamics. Here we use an unsupervised machine learning algorithm to identify structural heterogeneities in three archetypical glass formers—without using any dynamical information. In each system, the unsupervised machine learning approach autonomously designs a purely structural order parameter within a single snapshot. Comparing the structural order parameter with the dynamics, we find strong correlations with the dynamical heterogeneities. Moreover, the structural characteristics linked to slow particles disappear further away from the glass transition. Our results demonstrate the power of machine learning techniques to detect structural patterns even in disordered systems, and provide a new way forward for unraveling the structural origins of the slow dynamics of glassy materials.

## Abstract (web summary)

The origin of dynamical slowdown in disordered materials remains elusive, especially in the absence of obvious structural changes. Boattini et al. use unsupervised machine learning to reveal correlations between structural and dynamical heterogeneity in supercooled liquids.

## Introduction

The connection between structure and dynamics in supercooled liquids and glasses is one of the most intriguing puzzles in condensed matter physics. The conundrum: close to the glass transition, the dynamics slow down dramatically and become heterogeneous^1,2^ while the structure appears largely unperturbed. Largely unperturbed, however, is not the same as unperturbed, and many studies have attempted to identify slow local structures by exploiting dynamical information^3,4^. Unsupervised machine learning (UML) techniques may provide a novel way forward for shedding light on this problem.

Machine learning (ML) techniques are rapidly becoming a game-changer in the study of materials. Examples include speeding up computationally expensive calculations^5^, accurately distinguishing different crystal phases^6,7^, and even developing design rules for structural and material properties^8^. An exciting development is the design of UML algorithms that can autonomously classify particles based on patterns in their local environment^9–11^, even in disordered systems^12^. A key strength of these UML approaches is that they can find variations in local structure without any a priori knowledge of what might appear, opening the door to finding new, unanticipated structures.

The idea of an autonomous algorithm that picks out structural heterogeneities is a particularly appealing one in the context of supercooled liquids. In this field, the last few years have seen a frantic hunt for local structural features that can be interpreted as the underlying cause for dynamical heterogeneities. To this end, a number of studies have examined the prevalence and lifetimes of a large variety of locally favored structures^13,14^, correlated dynamics with local order parameters based on, e.g., tetrahedrality or packing efficiency^15–17^, and have looked at the dynamical effects of promoting specific local features^18–20^.

A major advance in correlating structure and dynamics was made through the use of supervised ML techniques. In particular, a number of studies have demonstrated that support-vector machines could be taught to recognize more mobile particles in several glass formers^21–24^. More recently, it was shown that even better dynamical predictions could be made using both convolutional neural networks and graph neural networks^25^. However, in order to train these algorithms, data linking structure to future dynamics had to be used. Methods that can autonomously detect purely structural heterogeneities offer an unbiased fresh look at the problem.

Here we show that a simple, efficient UML algorithm that we recently developed^10^ for detecting crystalline structure can be harnessed to detect structural heterogeneities in glasses. Our algorithm—which requires only a single snapshot as input—uses bond-order parameters (BOPs) to encode the local environments of the particles. Combining a neural network-based autoencoder with Gaussian mixture models, it then autonomously designs a structural order parameter capable of detecting the largest structural variation in the system.

We test the performance of the algorithm on three archetypical glass forming systems: binary hard spheres, Wahnström, and Kob–Andersen. These three model systems have been extensively studied in the context of fundamental glass formers, and have proven extremely valuable in unraveling many aspects of the glass transition (see e.g. refs. ^3,26,27^). Additionally, extensive past research has indicated that both binary hard spheres and the Wahnström model display a strong correlation between local structure and dynamical slowdown^3,28^, while these correlations are more nebulous for the Kob–Andersen model^28^. Collectively, these models provide an ideal playground for testing the ability of our UML technique to find local structural features in supercooled liquids.

## Results

### Setting up the UML algorithm

We construct configurations for our UML analysis by running molecular dynamics simulations of three glass formers inside the glassy regime. The glass formers we consider are all three-dimensional models and include binary hard spheres, Wahnström, and Kob–Andersen (see “Methods” for more details). We then select one equilibrated configuration in the glassy regime for each glass former to start our UML analysis.

**Fig. 1:**
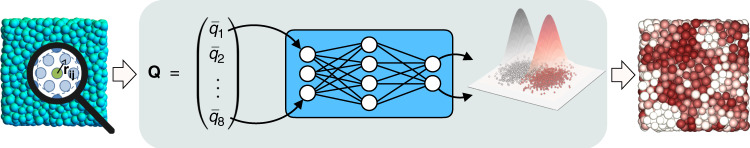
Schematic representation of the unsupervised machine learning method. In this method, the local environment of a particle is encoded in a vector (**Q**) of bond-order parameters, which is used as the input for an artificial neural network trained to reduce its dimensionality. The resulting distribution of particle environments in the lower dimension is clustered using a Gaussian mixture model. Finally, particles are assigned a probability of belonging to one of the two clusters, and colored accordingly.

The UML method we explore here is based on an algorithm we recently developed^10^ for detecting crystalline structures. As shown in Fig. 1, this analysis consists of three steps. First, the local environment of each particle is encoded into a vector of eight BOPs (see “Methods”). This local environment includes information regarding (approximately) the first two shells of neighbors. Secondly, an autoencoder is used to lower the dimensionality of this vector. The autoencoder is a neural network trained to reproduce its input as its output. This neural network is especially designed to contain a “bottleneck” with a lower dimensionality than the input vector, such that the network is forced to compress the information, and subsequently decompress it again. After training the autoencoder, we only retain the compression part of the network, and use it as our dimensionality reducer. Note that this algorithm allows for non-linear transformations to a lower dimension. Third, the particles are then grouped in this lower-dimensional space using Gaussian mixture models. A full description of this algorithm is given in the Supplementary Methods.

**Fig. 2:**
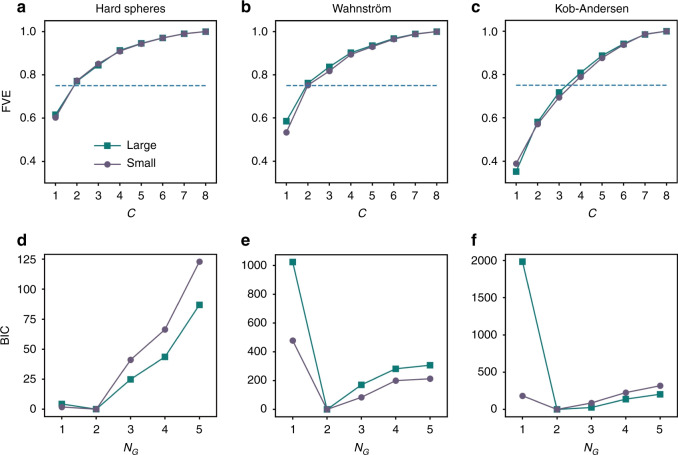
Determining UML parameters. **a**–**c** Fraction of variance explained (FVE) as a function of the dimension of the bottleneck, *c*, for large and small particles in the three glass formers. The dashed blue line corresponds to a threshold of FVE = 0.75. **d**–**f** Bayesian information criterion (BIC) as a function of the number of Gaussian components, *N*_*G*_, for large and small particles in the three systems. Note that for illustration purposes the BIC has been shifted to have a minimum value of BIC = 0. The three systems are, from left to right: hard spheres with packing fraction *η* = 0.58, size ratio *q* = 0.85, and composition *x*_*L*_ = 0.3, Wahnström at density ρ^*^ = 0.81 and temperature T^*^ = 0.7, and Kob–Andersen at density ρ^*^ = 1.2 and temperature T^*^ = 0.5.

This UML algorithm has two main parameters that need to be chosen: (i) the dimensionality of the bottleneck of the autoencoder *c* and (ii) the number of Gaussian components *N*_*G*_ used to fit the distribution in the lower-dimensional space. To choose the dimensionality of the bottleneck, we require that the autoencoder reproduces at least 75% of the variance of the input data. In Fig. 2a–c we show the fraction of variance explained (FVE, see “Methods”) by the autoencoder as a function of *c* for the chosen snapshots from all three models. Based on this analysis, we choose *c* = 2 for both the binary hard spheres and the Wahnström models, and *c* = 4 for the Kob–Andersen model. To determine the number of Gaussians *N*_*G*_, we measure the Bayesian Information Criterion (BIC, see Supplementary Methods) as a function of *N*_*G*_, and plot it for each snapshot in Fig. 2d–f. The optimum number of Gaussians corresponds to the minimum in the BIC analysis, which for all models is found at *N*_*G*_ = 2. As each Gaussian can be seen as generating a cluster in the data, this means that the UML identifies two clusters of particles for all three models.

In addition to these two parameters, the depth and width of the neural network forming the autoencoder can be varied. In this paper we always use a network geometry of one hidden layer of dimension 40 for both the encoder and the decoder parts of the network. In the Supplementary Note 1, we explore the behavior of our UML algorithm, with respect to, e.g., the dimensionality of the input vector, repeated network trainings, network structure, and the dimensionality of the bottleneck *c*. We find the algorithm to be largely robust with respect to changes in these parameters.

### Extracting a scalar order parameter *P*_red_

To encode the UML clustering into a single scalar order parameter, each particle is assigned a probability to belong to one of the two clusters. To this end, we label the two clusters with different colors—white and red, see Fig. 1. For each particle *i*, we then define *P*_red_(*i*) as the probability that the particle belongs to the red cluster based on the Gaussian mixture model (see “Methods”). This results in a single scalar order parameter between 0 and 1, with values near 0 indicating particles whose environment more closely matches the white cluster, and values near 1 indicating particles whose environment more closely matches the red cluster. Note that by definition this order parameter captures the largest structural heterogeneities in the system, as found by the UML approach. We would like to stress that in contrast with supervised ML studies of glasses^21,22^, our approach uses no dynamical information, and is trained on a single static snapshot for each system.

**Fig. 3:**
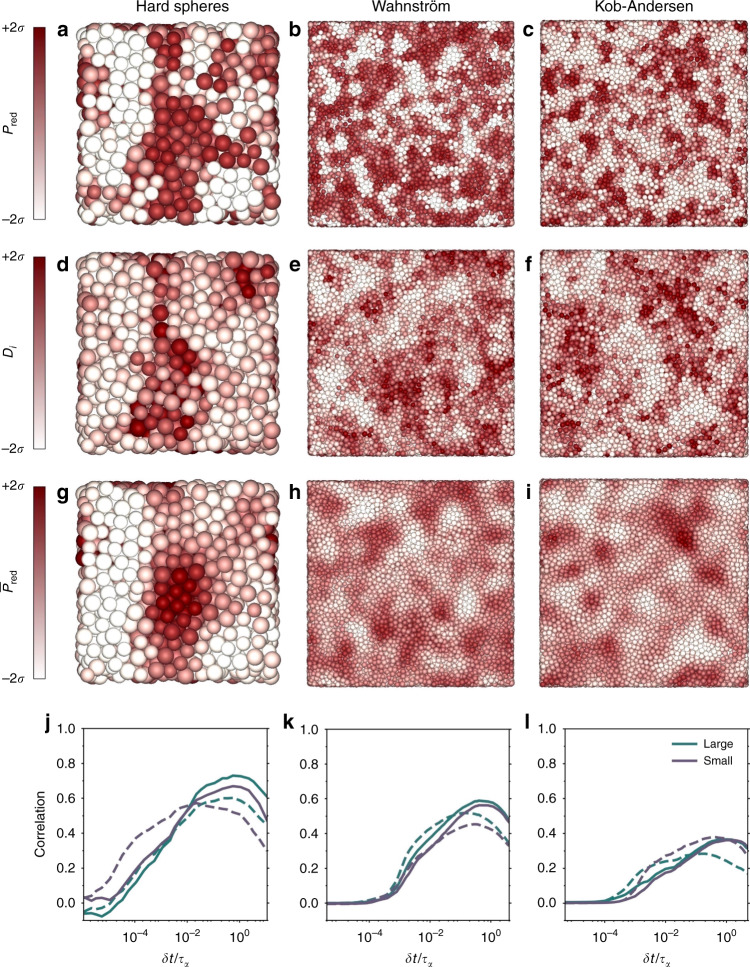
Structural analysis and correlations with dynamics in three archetypical glass formers. **a**–**c** Snapshots of different glassy models. From left to right: hard spheres, Wahnström, and Kob–Andersen at the same state points as Fig. 2. Particles are colored according to their membership probability *P*_red_ of belonging to a specific cluster identified by the machine learning approach. In particular, particles whose *P*_red_ is two or more standard deviations *σ* above the mean value are dark red, while particles with *P*_red_ more than two *σ* below the mean are colored white. **d**–**f** Same snapshots as **a**–**c**, but colored according to the dynamic propensity *D*_*i*_. **g**–**i** Same snapshots, colored according to the locally averaged membership probability ${\bar{P}}_{\text{red}}(i)$, where the averaging is over a local spherical neighborhood with a radius of two times the diameter of the spheres of species *A*. **j**–**l** Spearman’s rank correlation between the particles’ dynamic propensity *D*_*i*_ and either their membership probability *P*_red_(*i*) (dashed lines) or its local average, ${\bar{P}}_{\text{red}}(i)$ (solid lines).

Using this scalar order parameter, we analyze the three glassy configurations, and color each particle based on *P*_red_. The results are shown in Fig. 3a–c. In all three systems, the system shows clear structural heterogeneity, consisting of regions of both environments.

### Correlating *P*_red_ with dynamics

The question now is whether the structural variations detected by *P*_red_ are correlated with the dynamics. To probe this, we measure the dynamic propensity *D*_*i*_(*δt*) of particle *i*: a measure for how mobile particle *i* will be over the next time interval *δt* (see “Methods”), which has proven useful in supercooled liquids^15,16,29,30^. In Fig. 3j–l, we plot the Spearman’s correlation coefficient between *P*_red_ and *D*_*i*_(*δt*), as a function of the time interval *δt*. As one might expect, this correlation is weak both for very short time scales, where particles are simply rattling within their cages, and for long time scales where the system loses memory of its initial configuration. It peaks slightly below the structural relaxation time *τ*_*α*_, indicating that we have indeed identified structures connected to the structural relaxation.

To further investigate the correlation between the UML order parameter and the dynamics, in Fig. 3d–f, we color the particles according to their dynamic propensity, with *δt* chosen to correspond to the maximum in the correlation. Comparing to Fig. 3a–c, it is clear that regions of high dynamic propensity correspond to high values of *P*_red_, indicating that the particles identified as part of the red cluster also largely correspond to the faster particles in the system. The correlation can be further improved by averaging *P*_red_ over particles within a small local region, similar to what was found in previous studies^15,16,31^. This is shown in the snapshots in Fig. 3g–i and the solid lines in Fig. 3j–l, where we show the results for a local averaging radius *r*_c_ = 2*σ*. In all cases, the correlation between the averaged ${\bar{P}}_{{\rm{red}}}$ and *D*_*i*_ peaks very close to *τ*_*α*_. This is slightly later than the unaveraged version, likely because we are now looking at larger regions, which will take more time to rearrange. Note that here we resolved the inherent symmetry between red and white clusters, by always labeling as red the cluster that on average turns out to be faster.

As also found in previous work^28^, Kob–Andersen seems to be the model whose behavior is less well captured by our analysis. This might be related to the attraction that could induce heterogeneities over large length scales due to the proximity of a gas–liquid phase coexistence^32,33^. This kind of effect would not be fully captured by our (highly local) observables.

To summarize, in all cases the structural heterogeneities identified by our UML order parameter correlate significantly with the local dynamics. This leads to two intriguing questions: (i) are these structural correlations to the dynamics strong or weak in comparison to literature? and (ii) how does the method perform when compared to supervised learning algorithms specifically designed to predict dynamics?

With respect to the first question (i), in the case of binary hard spheres, previous literature has shown a strong correlation between local tetrahedrality and both global and local dynamics. At the same state point shown in Fig. 3 for binary hard spheres, the correlation between local tetrahedrality and local dynamics was shown to reach  ≈0.63 for small particles^15^. Our UML order parameter significantly exceeds this with a value of  ≈0.72. For Wahnström, and Kob–Andersen, Hocky et al.^28^ examined the correlation between locally favored structures and dynamic propensity. Although the state points are not exactly the same as ours, they found a slightly stronger correlation for Wahnström, and a slightly weaker one for Kob–Andersen.

With respect to the second question (ii), we will focus on the Kob–Andersen system where the most extensive data exist. In a recent article, Bapst et al.^25^ examined both a variety of physics-based methods for predicting propensity (based on soft modes^34^, the Debye–Waller factor^30^, or potential energy^29,35^), as well as state of the art supervised ML algorithms^21,25^. As shown in Supplementary Fig. 7, at the strongest supercooling ${\bar{P}}_{{\rm{red}}}$ (with a correlation of 0.4) correlates better than any of the physics-based methods (best correlation: 0.35), but worse than the supervised ML algorithms (range of correlations: 0.4–0.6). The higher correlations in the supervised algorithms were expected as these algorithms explicitly fit dynamic propensity as a function of structural features. However, in contrast to the UML algorithm, such fitting requires a large amount of data and computational effort.

### Variation of *P*_red_ with supercooling

**Fig. 4:**
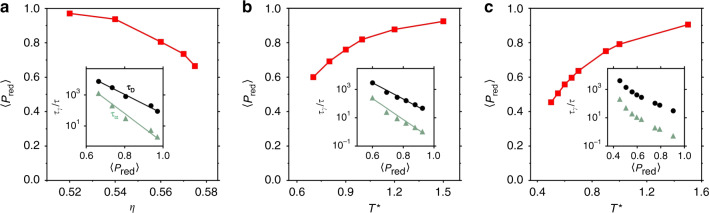
Structural order parameter as a function of supercooling. Mean membership probability $<{P}_{\text{red}}>$ for the three systems: **a** as a function of the packing fraction *η* for hard spheres, and as a function of the reduced temperature T^*^ for **b** Wahnström and **c** Kob–Andersen. The insets show the relation between $<{P}_{\text{red}}>$ and either the structural relaxation time *τ*_*α*_ (green triangles) or the diffusion time *τ*_D_ (black circles). In all cases, *P*_red_ is calculated using the UML approach trained at the highest degree of supercooling.

Thus far, we have shown that *P*_red_ is capable of predicting local variations in the dynamics in each of our systems. A natural next question is whether the same order parameter can be used globally, i.e. to capture the onset of dynamical arrest as the glass former is supercooled. This can be done by checking whether the “slow” structural group becomes more dominant as we move closer to the glass transition, similar to what was seen in, e.g., refs. ^15,23,36^. To confirm that the UML-designed order parameters are able to capture the onset of dynamical arrest without the need for retraining, we use the exact same UML order parameter trained on the snapshots of Fig. 3 on systems equilibrated at lower degrees of supercooling: lower packing fractions *η* for hard spheres, and higher temperatures T^*^ for the other two models. In Fig. 4, we plot 〈*P*_red_〉, defined as the globally averaged value of *P*_red_, as a function of the degree of supercooling for each glass former. In all cases, 〈*P*_red_〉 increases monotonically as the system moves out of the glassy regime. Hence, the structures we identify as white (slow) at strong supercooling disappear as we move away from the glass transition—clearly showing that the UML order parameter identifies local structures that are important for the dynamical slowdown. Interestingly, as shown in the insets in Fig. 4, the relationship between 〈*P*_red_〉 and the structural relaxation time is exponential for both the hard sphere and the Wahnström system.

### Predictive power as a function of supercooling

**Fig. 5:**
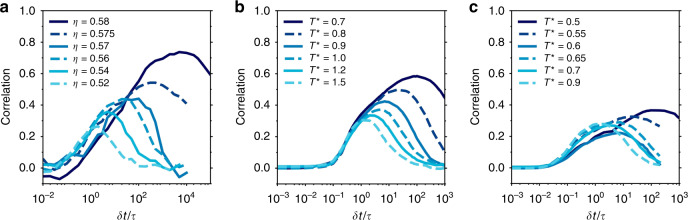
Correlation between structure and dynamics for different supercoolings. Correlation between the locally averaged ${\bar{P}}_{\rm{red}}$ and dynamic propensity for large particles (see Supplementary Note 3 and Supplementary Fig. 8 for small particles). Note that the averaging radius for ${\bar{P}}_{{\rm{red}}}$ is 2*σ*_*A*_ in all cases. In all cases, the UML approach is retrained on a snapshot from the system in question. The systems are **a** hard spheres, **b** Wahnström, and **c** Kob–Andersen.

It is well known that the dynamical heterogeneities of supercooled liquids become weaker and shift to shorter time scales as we move away from the glass transition. As a result, the correlation between structure and dynamics should also become weaker^3,15,16^. To test how the predictive power of our UML analysis depends on the degree of supercooling, we perform a new UML analysis on each of the glass formers at different packing fractions and temperatures. Specifically, for each state point we find a new projection and classification, and determine the correlation between ${\bar{P}}_{{\rm{red}}}$ and *D*_*i*_. Note that this is different from our analysis for Fig. 4 as there we used only a single-order parameter for each system. By retraining the order parameter, we correlate the dynamical heterogeneity with the largest structural variation that we can find at each individual state point. In Fig. 5 we show that indeed the correlations become weaker and shift to shorter times (along with *τ*_*α*_) as we move away from the glassy regime.

In Fig. 5 we have kept the local averaging radius *r*_c_ fixed at 2*σ*_*A*_ for the calculation of ${\bar{P}}_{{\rm{red}}}$. However, in practice, the maximum in the correlation depends on the choice of *r*_c_, as shown in the Supplementary Note 4. Specifically, the optimal value of *r*_*c*_ tends to increase with the degree of supercooling. It might be tempting to interpret this growth as a growing static length scale, a topic of significant debate in the glass community (see, e.g., refs. ^3,37–40^). However, it is important to realize that this optimum value is determined by using dynamical data (i.e. the correlation with the dynamic propensity). As such, the optimum in *r*_c_ should be seen as a dynamic length scale. In contrast, one can straightforwardly extract a static correlation length from our order parameter *P*_red_, by determining its autocorrelation function as a function of distance. As shown in the Supplementary Note 5, this method gives an essentially constant correlation length for each of our three systems, with little to no growth with increasing supercooling. Hence, our method does not yield a clearly growing, purely static length scale.

### Characterizing the local structure of the different clusters

**Fig. 6:**
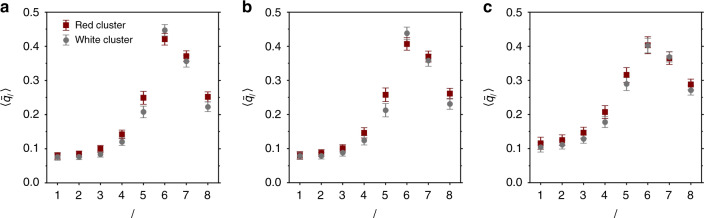
Variation of bond-order with *P*_red_. Mean values of the BOPs for large particles with *P*_red_ > 0.5 (red) and *P*_red_ < 0.5 (white) in **a** hard spheres, **b** Wahnström, and **c** Kob–Andersen systems. The state points are the same as in Fig. 2. Bars are the standard deviations. The results for small particles are similar, see Supplementary Fig. 12.

As the UML is based on a description of local environments in terms of BOPs^41^, a natural question to ask is how the two identified clusters differ in terms of their BOPs. In Fig. 6, we plot the mean value of all BOPs *q*_1_, … , *q*_8_ for both red (more mobile, *P*_red_ > 0.5) and white clusters (*P*_red_ < 0.5) for the three snapshots depicted in Fig. 3. Perhaps surprisingly, we do not observe dramatic differences in the average BOPs of the two clusters. The small variations that are seen, however, exhibit a few notable trends. Specifically for all three models, *q*_2_, *q*_3_, *q*_4_, *q*_5_, and *q*_8_ are higher in the red, more mobile cluster. Additionally, for both hard spheres and Wahnström, *q*_6_ is the highest in the slow cluster; note that *q*_6_ is often connected to close packed crystal structures like face-centered cubic and hexagonal-close packed.

**Fig. 7:**
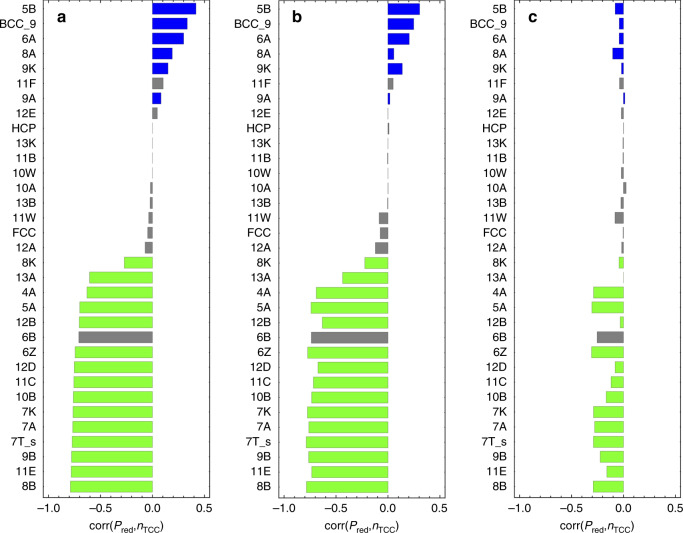
Correlation between *P*_red_ and TCC clusters. Correlation between the membership probability *P*_red_ of a particle and the number of TCC clusters of a given type the particle is involved in, for the three investigated systems. From left to right: **a** hard spheres, **b** Wahnström, and **c** Kob–Andersen, at the same state points as Fig. 2. The clusters are sorted based on their correlation in the hard-sphere model. Green bars indicate clusters that consist of one or more tetrahedral subclusters, while blue bars indicate clusters that consist of one or more square pyramidal subclusters. Gray clusters contain neither (cluster 6B is a single pentagonal pyramid), or both.

As a second avenue for differentiating the local structure in each group we use topological cluster classification (TCC)^42^. This algorithm detects a set of pre-defined clusters corresponding to low-energy (or high-packing) structures in a few model systems. As shown in Fig. 7, we find that for the hard sphere and Wahnström systems, *P*_red_ negatively correlates strongly with local structures built up out of one or more tetrahedra, while it positively correlates with TCC clusters built from square pyramids. For the Kob–Andersen mixture, *P*_red_ still negatively correlates best with tetrahedral environments, but correlations are significantly weaker. Interestingly, TCC detects essentially no clusters that correlate positively with *P*_red_, suggesting that these particles have local environments not detected by TCC—indicating that our UML is picking up on structures not included in the low-energy (or high-packing) structures built into TCC. This is one area where the UML approach shines: it is not restricted by a priori assumptions about the features that are considered in the clustering.

## Discussion

Overall, our results are consistent with the idea that at least part of the dynamical slowdown is driven by the emergence of a set of locally favored structures, in agreement with earlier observations on these models (see, e.g., refs. ^19,43,44^). As the system is pushed closer to the glass transition, these structures become more stable, due to a more favorable local packing or potential energy, with a profound impact on both local and global dynamics^3^. What is intriguing is the observation that this variation in local structure can be effectively captured by a one-dimensional machine-learned order parameter, and that this structural order parameter strongly correlates with the dynamics.

Interestingly, our Gaussian clustering approach indicates that the structural features of all of our systems, once projected onto the lower-dimensional space, are best described by two Gaussians. This is consistent with a previously proposed picture where supercooled liquids can be interpreted as a mixture of two competing structural populations^20^. Note that a similar two-state picture has been extremely successful in understanding the glassy behavior of supercooled water^31,45^, where the competing local structures are ostensibly linked to different thermodynamic phases at extreme supercooling.

The UML analysis used here is both extremely simple to implement as well as very efficient to run. The input is a single snapshot, and the analysis involves standard BOP calculations, fitting a small neural network-based autoencoder, and Gaussian mixture models; all of which are fast, standard methods that are commonly available in open-source libraries. The total analysis costs only a few minutes of computational time. While the simplicity of this algorithm is one of its main strengths, this also implies that there are many potential routes towards expanding this method—for instance, adding information about the local density to the local descriptors, and/or projecting the information onto higher dimensional order parameters. Such additions might increase the accuracy, although likely at the cost of the simplicity and speed. Already, a number of UML techniques exist that classify particles based on local structure^9–12^, using different definitions of local structures, and different approaches for classification. It will be interesting to see which of these performs best in purely heterogeneous environments like glasses.

In conclusion, we have demonstrated that a simple and fast autoencoder-based UML approach is a powerful tool in the development of new *structural* order parameters in supercooled liquids. We demonstrated that the structural heterogeneities captured by this order parameter are strongly correlated with the dynamical heterogeneities in all three glass formers studied here, creating a new way forward for unraveling the microscopic origins of dynamical slowdown in supercooled liquids.

## Methods

### Models

We consider three model glass formers in three dimensions: binary hard spheres, Wahnström^26^, and Kob–Andersen^27^. Both Wahnström and Kob–Andersen are binary mixtures of Lennard–Jones (LJ) particles.

The binary hard-sphere model we consider is a mixtures of 30% large *A* particles and 70% small *B* particles, with size ratio *σ*_*B*_/*σ*_*A*_ = 0.85. The mode coupling theory (MCT) packing fraction for this model is *η*_MCT_ = 0.584 (see Supplementary Note 7).

The Wahnström model^26^ is an equimolar (50–50%) mixture of *A* and *B* particles. The LJ interaction strength between all pairs of particles is identical (*ϵ*_*AA*_ = *ϵ*_*AB*_ =  *ϵ*_*BB*_), but the *B* particles are slightly larger than the *A* particles (*σ*_*BB*_ = 1.2*σ*_*AA*_ and *σ*_*AB*_ = 1.1*σ*_*AA*_). The LJ potential is truncated and shifted at the minimum in the potential, such that the interactions are purely repulsive. The MCT temperature is *k*_B_*T*_MCT_/*ϵ*_*AA*_ = 0.652 (see Supplementary Note 7).

The Kob–Andersen model^27^ is a non-additive mixture of 80% (large) *A* particles and 20% (small) *B* particles. The interaction parameters are *σ*_*BB*_ = 0.88*σ*_*AA*_, *σ*_*AB*_  = 0.8*σ*_*AA*_, *ϵ*_*BB*_ = 0.5*ϵ*_*AA*_, and *ϵ*_*AB*_ = 1.5*ϵ*_*AA*_. The LJ potential is truncated and shifted at a cutoff distance *r*_c,*ij*_ = 2.5*σ*_*ij*_ (where *i*, *j* ∈ {*A*, *B*}), such that the attractive part of the potential is retained. The MCT temperature for this model is *k*_B_*T*_MCT_/*ϵ*_*AA*_ = 0.42 (see Supplementary Note 7).

For both Wahnström and Kob–Andersen, we define the reduced number density ${\rho }^{* }=\rho {\sigma }_{AA}^{3}$ and reduced temperature T^*^ = *k*_B_*T*/*ϵ*_*AA*_, with *k*_B_ Boltzmann’s constant.

### Simulations

For all models, we use molecular dynamics simulations in the canonical ensemble. In the case of hard spheres, the simulations are performed using an event-driven approach. For Wahnström and Kob–Andersen, we use the simulation package LAMMPS^46^.

Dynamic propensities are calculated as an isoconfigurational ensemble average of the absolute displacement of each particle. In other words, we perform at least 32 independent simulations starting from the same initial configuration, but with randomly chosen velocities for all particles. The dynamic propensity of particle *i* after a time interval *δt* is then defined as

1 $${D}_{i}(\delta t)={\langle \left|{{\bf{r}}}_{i}(\delta t)-{{\bf{r}}}_{i}(0)\right|\rangle }_{{\rm{c}}},$$

where **r**_*i*_(*t*) is the position of particle *i* at time *t*, and the average is taken over the independent runs.

In order to obtain the relaxation time *τ*_*α*_, we calculate the self-intermediate scattering function (ISF) for the Wahnström and the Kob–Andersen systems, and the total intermediate scattering function for the hard spheres:

2 $$F(q,t)=\frac{\left\langle{\sum }_{j,k}\exp \left\{i{\bf{q}}\left[{{\bf{r}}}_{j}(t)-{{\bf{r}}}_{k}(0)\right]\right\}\right\rangle}{\left\langle{\sum }_{j,k}\exp \left\{i{\bf{q}}\left[{{\bf{r}}}_{j}(0)-{{\bf{r}}}_{k}(0)\right]\right\}\right\rangle},$$

where **r**_*i*_ is the position of particle *i* and **q** is a wave vector. We calculate the ISF at an inverse wavelength *q* = ∣**q**∣ corresponding to the first peak of the structure factor. After that, we fit the long-time decay of the ISF with a stretched exponential function $\gamma \exp \left[-{(t/{\tau }_{\alpha })}^{\beta }\right]$, where *γ*, *β,* and the relaxation time *τ*_*α*_ are fit parameters.

### Local environment description

To characterize the local environment of each particle, we use an averaged version of the local BOPs introduced by Steinhardt et al.^41^. First, we define for any given particle *i* the complex quantities

3 $${q}_{lm}(i)=\frac{1}{{N}_{b}(i)}\sum _{j\in {{\mathcal{N}}}_{b}(i)}{Y}_{l}^{m}({{\bf{r}}}_{ij}),$$

where ${Y}_{l}^{m}({{\bf{r}}}_{ij})$ are the spherical harmonics of order *l*, with *m* an integer that runs from *m* = −*l* to *m* = +*l*. Additionally, **r**_*ij*_ is the vector from particle *i* to particle *j*, and ${{\mathcal{N}}}_{b}(i)$ is the set of nearest neighbors of particle *i*, which we will define later. Note that ${{\mathcal{N}}}_{b}(i)$ contains *N*_*b*_(*i*) particles. Then, the rotationally invariant BOPs, *q*_*l*_, are defined as^41^

4 $${q}_{l}(i)=\sqrt{\frac{4\pi }{2l+1}\mathop{\sum }\limits_{m=-l}^{l}| {q}_{lm}(i){| }^{2}}.$$

Finally, we define an average ${\bar{q}}_{l}(i)$ as

5 $${\bar{q}}_{l}(i)=\frac{1}{{N}_{b}(i)+1}\left[{q}_{l}(i)+\sum _{k\in {{\mathcal{N}}}_{b}(i)}{q}_{l}(k)\right].$$

Note that by taking this average, ${\bar{q}}_{l}(i)$ depends on the positions of not only the nearest neighbor shell of particles but also the second neighbor shell. Additionally, the quantities in Eq. (5) differ from the averaged BOPs introduced by Lechner and Dellago^47^ where first the averaging is performed on the non-rotational-invariant *q*_*lm*_, and then rotational-invariant quantities are built.

Our description of the local environment of particle *i* consists of an eight-dimensional vector,

6 $${\bf{Q}}(i)=(\{{\bar{q}}_{l}(i)\}),$$

with *l* ∈ [1, 8].

The set of nearest neighbors of each particle is identified with a parameter-free criterion called SANN (solid angle nearest neighbor)^48^ for the hard spheres and Wahnström models. In this approach, an effective individual cutoff radius, *r*_c_(*i*), is found for every particle *i* in the system based on its local environment. This method is not inherently symmetric, i.e., *j* might be a neighbor of *i* while *i* is not a neighbor of *j*. However, symmetry can be enforced by either adding *j* to the neighbors of *i* or removing *i* from the neighbors of *j*. In this study, we applied the latter solution. For the Kob–Andersen mixture, we obtained better results with a fixed cutoff radius (see Supplementary Note 8 for a comparison).

### Unsupervised ML

The UML approach used here follows the method outlined in ref. ^10^. A detailed description is provided in the Supplementary Methods.

### Fraction of variance explained

The optimal number of nodes in the bottleneck layer of the autoencoder, *c*, is determined by computing the FVE by the reconstruction,

7 $$\,{\text{FVE}}\,=1-\frac{\mathop{\sum }\nolimits_{i = 1}^{N}{\left\Vert {\bf{Q}}(i)-{\hat{\bf{Q}}}(i)\right\Vert }^{2}}{\mathop{\sum }\nolimits_{i = 1}^{N}{\left\Vert {\bf{Q}}(i)-{\bar{{\bf{Q}}}}\right\Vert }^{2}},$$

where $\bar{{\bf{Q}}}$ is the mean input vector and *N* is the number of particles. To choose *c* we require that this fraction is at least 75%.

### Definition of *P*_red_

As described in the main text, for all three models, after using the UML method to reduce the dimensionality of the data, we then cluster the data using Gaussian mixture models. In all cases, the optimum number of clusters was found to be two, which we label red and white. We then used this clustering to create a scalar order parameter *P*_red_ which quantifies the local structure around the particles as follows:

8 $${P}_{{\rm{red}}}=\frac{{g}_{{\rm{red}}}}{{g}_{{\rm{white}}}+{g}_{{\rm{red}}}},$$

where *g*_white(red)_ is the value of the fitted Gaussian peak associated with the white (red) cluster. Note that by definition *P*_white_ + *P*_red_ = 1. Hence, as we go from the white peak to the red peak and beyond (where *g*_white_ is very small), *P*_red_ monotonically increases. Note that even though dynamics are not used in this analysis, a posteriori we always assigned the label red to the cluster with the most mobile particles.

### TCC analysis

To correlate *P*_red_ with locally favored structures in the three model systems, we use the TCC algorithm^42^. Specifically, we count for each particle the number of clusters it is involved in of each type detected by the algorithm. We then calculate the correlation between the number of clusters of a given type a particle is a part of, and *P*_red_. Note that in its original form, TCC does not accurately count simple clusters (specifically tetrahedra, square pyramids, and pentagonal pyramids) which are subsumed into larger combinations of such clusters. Here we have adapted the algorithm to correct for this choice.

## Supplementary information

Supplementary Information

## Data Availability

The data associated with this research is available upon request.

## Code availability

The simulation and analysis codes associated with this research are available upon request.

## References

[1] Ediger MD (2000). Spatially heterogeneous dynamics in supercooled liquids. Annu. Rev. Phys. Chem..

[2] Berthier, L., Biroli, G., Bouchaud, J.-P., Cipelletti, L. & van Saarloos, W. *Dynamical Heterogeneities in Glasses, Colloids, and Granular Media*, Vol. 150 (OUP, Oxford, 2011).

[3] Royall CP, Williams SR (2015). The role of local structure in dynamical arrest. Phys. Rep..

[4] Tanaka H, Tong H, Shi R, Russo J (2019). Revealing key structural features hidden in liquids and glasses. Nat. Rev. Phys..

[5] Behler J, Parrinello M (2007). Generalized neural-network representation of high-dimensional potential-energy surfaces. Phys. Rev. Lett..

[6] Geiger P, Dellago C (2013). Neural networks for local structure detection in polymorphic systems. J. Chem. Phys..

[7] Boattini E, Ram M, Smallenburg F, Filion L (2018). Neural-network-based order parameters for classification of binary hard-sphere crystal structures. Mol. Phys..

[8] Butler KT, Davies DW, Cartwright H, Isayev O, Walsh A (2018). Machine learning for molecular and materials science. Nature.

[9] Reinhart WF, Long AW, Howard MP, Ferguson AL, Panagiotopoulos AZ (2017). Machine learning for autonomous crystal structure identification. Soft Matter.

[10] Boattini E, Dijkstra M, Filion L (2019). Unsupervised learning for local structure detection in colloidal systems. J. Chem. Phys..

[11] Spellings M, Glotzer SC (2018). Machine learning for crystal identification and discovery. AIChE J..

[12] Paret J, Jack RL, Coslovich D (2020). Assessing the structural heterogeneity of supercooled liquids through community inference. J. Chem. Phys..

[13] Malins A, Eggers J, Royall CP, Williams SR, Tanaka H (2013). Identification of long-lived clusters and their link to slow dynamics in a model glass former. J. Chem. Phys..

[14] Leocmach M, Tanaka H (2012). Roles of icosahedral and crystal-like order in the hard spheres glass transition. Nat. Commun..

[15] Marín-Aguilar S, Wensink HH, Foffi G, Smallenburg F (2020). Tetrahedrality dictates dynamics in hard sphere mixtures. Phys. Rev. Lett..

[16] Tong H, Tanaka H (2018). Revealing hidden structural order controlling both fast and slow glassy dynamics in supercooled liquids. Phys. Rev. X.

[17] Tong H, Tanaka H (2019). Structural order as a genuine control parameter of dynamics in simple glass formers. Nat. Commun..

[18] Taffs J, Royall CP (2016). The role of fivefold symmetry in suppressing crystallization. Nat. Commun..

[19] Marín-Aguilar S, Wensink HH, Foffi G, Smallenburg F (2019). Slowing down supercooled liquids by manipulating their local structure. Soft Matter.

[20] Speck T, Malins A, Royall CP (2012). First-order phase transition in a model glass former: coupling of local structure and dynamics. Phys. Rev. Lett..

[21] Cubuk ED (2015). Identifying structural flow defects in disordered solids using machine-learning methods. Phys. Rev. Lett..

[22] Schoenholz SS, Cubuk ED, Sussman DM, Kaxiras E, Liu AJ (2016). A structural approach to relaxation in glassy liquids. Nat. Phys..

[23] Schoenholz SS, Cubuk ED, Kaxiras E, Liu AJ (2017). Relationship between local structure and relaxation in out-of-equilibrium glassy systems. Proc. Natl Acad. Sci. USA.

[24] Landes FP, Biroli G, Dauchot O, Liu AJ, Reichman DR (2020). Attractive versus truncated repulsive supercooled liquids: the dynamics is encoded in the pair correlation function. Phys. Rev. E.

[25] Bapst V (2020). Unveiling the predictive power of static structure in glassy systems. Nat. Phys..

[26] Wahnström G (1991). Molecular-dynamics study of a supercooled two-component Lennard-Jones system. Phys. Rev. A.

[27] Kob W, Andersen HC (1995). Testing mode-coupling theory for a supercooled binary Lennard-Jones mixture I: the van Hove correlation function. Phys. Rev. E.

[28] Hocky GM, Coslovich D, Ikeda A, Reichman DR (2014). Correlation of local order with particle mobility in supercooled liquids is highly system dependent. Phys. Rev. Lett..

[29] Berthier L, Jack RL (2007). Structure and dynamics of glass formers: predictability at large length scales. Phys. Rev. E.

[30] Widmer-Cooper A, Harrowell P (2006). Predicting the long-time dynamic heterogeneity in a supercooled liquid on the basis of short-time heterogeneities. Phys. Rev. Lett..

[31] Shi R, Russo J, Tanaka H (2018). Origin of the emergent fragile-to-strong transition in supercooled water. Proc. Natl Acad. Sci. USA.

[32] Sastry S (2000). Liquid limits: Glass transition and liquid-gas spinodal boundaries of metastable liquids. Phys. Rev. Lett..

[33] Berthier L, Tarjus G (2009). Nonperturbative effect of attractive forces in viscous liquids. Phys. Rev. Lett..

[34] Widmer-Cooper A, Perry H, Harrowell P, Reichman DR (2008). Irreversible reorganization in a supercooled liquid originates from localized soft modes. Nat. Phys..

[35] Doliwa B, Heuer A (2003). What does the potential energy landscape tell us about the dynamics of supercooled liquids and glasses?. Phys. Rev. Lett..

[36] Royall CP, Williams SR, Ohtsuka T, Tanaka H (2008). Direct observation of a local structural mechanism for dynamic arrest. Nat. Mater..

[37] Karmakar S, Dasgupta C, Sastry S (2009). Growing length and time scales in glass-forming liquids. Proc. Natl Acad. Sci. USA.

[38] Berthier L, Biroli G, Bouchaud J-P, Tarjus G (2019). Can the glass transition be explained without a growing static length scale?. J. Chem. Phys..

[39] Wyart M, Cates ME (2017). Does a growing static length scale control the glass transition?. Phys. Rev. Lett..

[40] Sausset F, Tarjus G (2010). Growing static and dynamic length scales in a glass-forming liquid. Phys. Rev. Lett..

[41] Steinhardt PJ, Nelson DR, Ronchetti M (1983). Bond-orientational order in liquids and glasses. Phys. Rev. B.

[42] Malins A, Williams SR, Eggers J, Royall CP (2013). Identification of structure in condensed matter with the topological cluster classification. J. Chem. Phys..

[43] Turci F, Speck T, Royall CP (2018). Structural-dynamical transition in the Wahnström mixture. Eur. Phys. J. E.

[44] Crowther P, Turci F, Royall CP (2015). The nature of geometric frustration in the Kob-Andersen mixture. J. Chem. Phys..

[45] Caupin F, Anisimov MA (2019). Thermodynamics of supercooled and stretched water: unifying two-structure description and liquid-vapor spinodal. J. Chem. Phys..

[46] Plimpton S (1995). Fast parallel algorithms for short-range molecular dynamics. J. Comput. Phys..

[47] Lechner W, Dellago C (2008). Accurate determination of crystal structures based on averaged local bond order parameters. J. Chem. Phys..

[48] Van Meel JA, Filion L, Valeriani C, Frenkel D (2012). A parameter-free, solid-angle based, nearest-neighbor algorithm. J. Chem. Phys..

