# Supplementary material for: Autonomously revealing hidden local structures in supercooled liquids
Source: Nat Commun. 2020 Oct 30;11:5479. doi: 10.1038/s41467-020-19286-8 (PMC7603397; doi:10.1038/s41467-020-19286-8)
Supplement: Supplementary file 1 — Supplementary Information [file 41467_2020_19286_MOESM1_ESM.pdf]

Supplementary Information
Autonomously revealing hidden local structures in supercooled liquids

Emanuele Boattini[1], Susana Marín-Aguilar[2], Saheli Mitra[2], Giuseppe Foffi[2], Frank Smallenburg[2], Laura Filion[1]
[1] *Soft Condensed Matter, Debye Institute of Nanomaterials Science, Utrecht University, Utrecht, Netherlands*
[2] *Université Paris-Saclay, CNRS, Laboratoire de Physique des Solides, 91405 Orsay, France*

CONTENTS

Supplementary Methods 2
A. Nonlinear dimensionality reduction using neural-network-based autoencoders 2
B. Clustering 3
C. Dimensionality reduction in the Wahnström mixture 3

Supplementary Note 1: Method Robustness 4
A. Different trainings 4
B. Autoencoder architecture 4
C. Bottleneck dimensionality 4
D. Input dimensionality 6

Supplementary Note 2: Comparison to supervised methods for linking structure and dynamics 6

Supplementary Note 3: Dynamic propensity correlations for small particles 7

Supplementary Note 4: Local averaging of the structural order parameter 7
A. Correlations for different cutoff radii 7
B. Length scale 8

Supplementary Note 5: Correlations in the trained order parameters 8

Supplementary Note 6: Bond order parameters in the two clusters of small particles 9

Supplementary Note 7: Mode coupling theory fits 9

Supplementary Note 8: SANN vs. cutoff in Kob-Andersen 10

Supplementary Note 9: Pearson vs. Spearman correlation 11

Supplementary References 11

SUPPLEMENTARY METHODS

In this paper, we employ an unsupervised machine learning (UML) method to classify particles into different groups based on their local environment. As mentioned in the Methods section, we describe the local environment of particle i in terms of a vector $\mathbf{Q}(i)$ of bond order parameters. This vector has length $d = 8$. As clustering particles with similar environments is difficult in such a high-dimensional space, we reduce the dimensionality of this vector using a neural-network-based autoencoder. A sketch of such an autoencoder is shown in Supplementary Figure 1.

Essentially, the autoencoder is a neural network trained to reproduce its input as its output. The neural network is especially designed to contain a "bottleneck" with a dimensionality lower than the input and output vectors, such that the network is forced to compress the information, and subsequently decompress it again. After training the autoencoder (which can be done on a single simulation snapshot), we only retain the encoding part of the network, and use it as our dimensionality reducer (as shown in Fig. 1 of the main text). This assigns to each particle a lower-dimensional vector which can be used to group particles with similar environments.

In order to cluster together similar environments in the low-dimensional subspace found by the encoder, we use Gaussian mixture models (GMMs) as implemented in scikit-learn[1]. This allows us to separate the particles into separate clusters which differ by their local structure. In all cases, we found a separation into two clusters to be optimal. Based on the resulting classification, we assign to each particle a probability $P_{\rm red}$ of belonging to a specific one of the two clusters. Note that in principle, the two clusters carry no inherent meaning. However, in order to make our figures consistent, we choose (using hindsight) the red cluster to correspond to the more mobile particles in the system.

In the following, we describe in more detail both the dimensionality reduction and the clustering procedures, which closely follow the method introduced in Ref. [2]. Note that, for all systems analysed in this work, we perform a separate analysis for the two species of particles.

A. Nonlinear dimensionality reduction using neural-network-based autoencoders

In order to reduce the dimensionality, i.e. extract the relevant information, of the vectors $\mathbf{Q}(i)$, we use neural-network-based autoencoders [3–7]. An autoencoder is a neural network that is trained to perform the identity mapping, where the network inputs, $\mathbf{Q}(i) \in \mathbb{R}^d$, are approximately reproduced at the output layer, $\hat{\mathbf{Q}}(i) \in \mathbb{R}^d$. The network may be viewed as consisting of two parts: an encoder network, which performs a nonlinear projection of the input data onto a low-dimensional subspace (the bottleneck), $\mathbf{Y}(i) \in \mathbb{R}^c$ with $c < d$, and a decoder network that attempts to reconstruct the input data from the low-dimensional projection. This architecture is represented in Supplementary Figure 1.

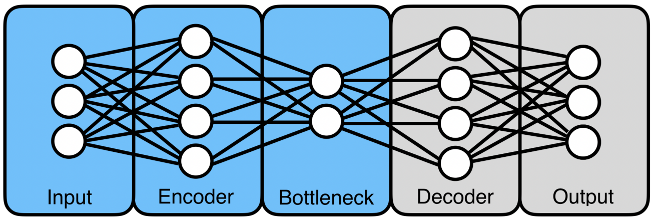

Supplementary Figure 1: Architecture of a neural-network based autoencoder. The encoder network (highlighted in blue) finds a low dimensional representation of the input, from which the decoder reconstructs an approximation of the input as output.

In this paper, we train the autoencoder using the data associated with the N different local environments from a single snapshot, where N is the number of particles in the simulation. By training the autoencoder to perform the input reconstruction task over this set of N training examples, the encoder is forced to learn a low-dimensional nonlinear projection that preserves the most relevant features of the data and from which the higher-dimensional inputs can be approximately reconstructed by the decoder.

The number of input and output nodes, d, is specified by the dimension of the input vectors. Nonlinearity is achieved by providing both the encoder and the decoder with a fully-connected hidden layer with a nonlinear activation function. In this work, we set the number of nodes in the hidden layers to $5d$ and use a hyperbolic tangent as the activation function. For the bottleneck and output layers, instead, a linear activation function is used.

The internal parameters of the autoencoder, i.e. weights $\mathbf{W} \equiv \{w_j\}$ and biases $\mathbf{B} \equiv \{b_k\}$, which are initialized with the normalized initialization proposed by Xavier in Ref. [8], are optimized during the training by iteratively minimizing the reconstruction error of the input data over a training set of N training examples. Specifically, we consider the mean squared error with the addition of a weight decay regularization term[7] to control the magnitude of the network weights

$$E(\mathbf{W}, \mathbf{B}; \{\mathbf{Q}(i)\}) = \frac{1}{N}\sum_{i=1}^{N} \left\| \mathbf{Q}(i) - \hat{\mathbf{Q}}(i) \right\|^2 + \lambda \sum_{j=1}^{M} w_j^2, \tag{1}$$

where M is the total number of weights, whose value depends on the dimension of the network, and we set $\lambda = 10^{-5}$. The function in Supplementary Equation 1 is minimized using mini-batch stochastic gradient descent with momentum[7, 9, 10].

The optimal number of nodes in the bottleneck layer, c, which defines the unknown relevant dimensionality of the input data, can be determined by computing the fraction

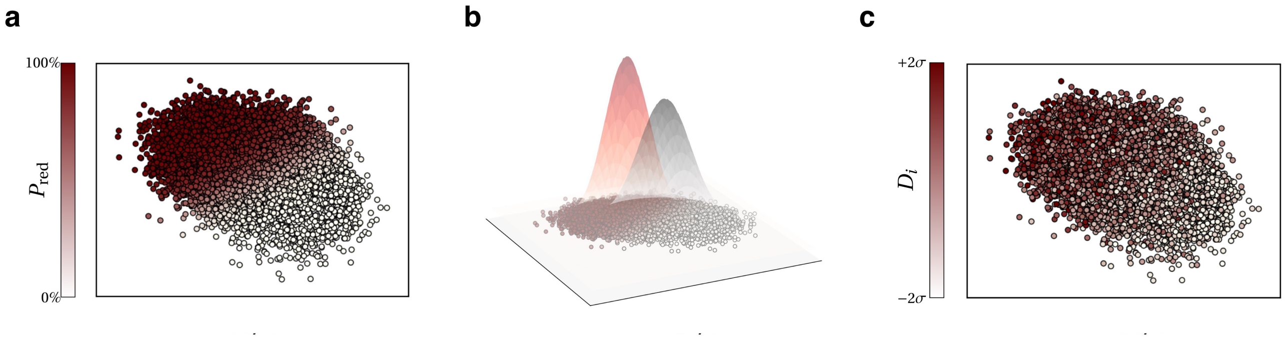

Supplementary Figure 2: **a** Scatterplot of the local descriptors of all large particles in the Wahnström after dimensionality reduction. The points are colored according to their value of $P_{\rm red}$. **b** Same scatterplot, with a representation of the two Gaussians found by the clustering. **c** Same scatterplot, but with points colored according to the dynamic propensity measured at the time with the maximum correlation. Note that the particles are colored according to their deviation from the mean dynamic propensity, in units of the standard deviation σ.

of variance explained (FVE) by the reconstruction,

$$\mathrm{FVE} = 1 - \frac{\sum_{i=1}^{N} \left\| \mathbf{Q}(i) - \hat{\mathbf{Q}}(i) \right\|^2}{\sum_{i=1}^{N} \left\| \mathbf{Q}(i) - \bar{\mathbf{Q}} \right\|^2}, \qquad (2)$$

where $\bar{\mathbf{Q}}$ is the mean input vector. Fig. 2**a** of the main text shows the FVE as a function of c for the three systems analyzed in this work. Here, we choose the optimal value of c as the smallest value for which the FVE is at least 0.75, i.e. at least 75% of the variance of the original data is explained by the autoencoder's reconstruction. As shown in Fig. 2**a**, this condition is satisfied with a choice of $c = 2$ both for the hard sphere and Wahnström systems, while a dimensionality of $c = 4$ was necessary for the Kob-Andersen system.

Once the autoencoder is trained, the encoder network (highlighted in blue in Supplementary Figure 1) alone is retained in order to perform the nonlinear mapping of the input vectors $\mathbf{Q}(i)$ onto the low-dimensional subspace defined by the bottleneck layer, $\mathbf{Y}(i)$.

B. Clustering

GMM is a probabilistic model that assumes that the observed data are generated from a mixture of a finite number of Gaussian distributions with unknown parameters. The optimal values of these parameters are found iteratively with the expectation-maximization (EM) algorithm[11] in order to create a probability density function that agrees well with the distribution of the data. The number of Gaussian components in the mixture, N_G, is usually found by minimizing the Bayesian information criterion (BIC)[12], which measures how well a GMM fits the observed data while penalizing models with many parameters to prevent overfitting. Fig. 2**b** of the main text shows that the BIC has a minimum at $N_G = 2$ for all the three systems and for both species of particles, indicating that a separation into two clusters is optimal for these systems.

The output of a trained GMM is a list of probabilities, $P_j(i)$, corresponding to the posterior probabilities of the i-th observation to arise from the j-th component in the mixture model. Here, we arbitrarily label the two clusters as "red" and "white" and refer to these probabilities as $P_{\rm red}(i)$ and $P_{\rm white}(i) = 1 - P_{\rm red}(i)$.

C. Dimensionality reduction in the Wahnström mixture

In order to demonstrate the effect of the dimensionality reduction, we show in Supplementary Figure 2**a** a scatterplot of all local environments of large particles in the Wahnström system at density $\rho^* = 0.81$ and temperature $T^* = 0.7$. For each particle, the encoding part of the autoencoder has been used to reduce the dimensionality of the vector $\mathbf{Q}$ to two dimensions. As a result, the information retained by the autoencoder can be plotted as a point in 2D space for each particle. Using the Gaussian Mixture Model, we classify this set of points into two clusters (white and red), corresponding to two Gaussian distributions of points (Supplementary Figure 2**b**). The connection to dynamics can also be clearly visualized in this reduced space, by coloring particles according to their dynamic propensity (Supplementary Figure 2**c**). This leads to a clear gradient in coloring, indicating that the faster particles (red) lie predominately in the top left cluster, while the slow particles lie in the bottom right one.

SUPPLEMENTARY NOTE 1: METHOD ROBUSTNESS

A. Different trainings

The training of the autoencoder is performed with a stochastic optimization algorithm and, as a consequence, the learned projection of the input data differs for every particular training. Although the FVE is an excellent indication of the quality of the learned projection, it is not a priory clear how strongly the final results depend on the particular projection found by the encoder.

To explore this, we performed multiple times the analysis for the large particles of the Wahnström system at reduced density $\rho^* = 0.81$ and reduced temperature $T^* = 0.7$. Every time, we trained a new autoencoder (with the same architecture and a different random seed), and subsequently performed a new clustering. Finally, we compared the Spearman's rank correlation between the dynamic propensities of the particles and their membership probabilities $P_{\rm red}$ obtained for different trainings of the UML method.

Supplementary Figure 3 shows that the results obtained for three different trainings are essentially the same, indicating that the method is robust to changes in the training.

B. Autoencoder architecture

In all the analysis performed in this work, we used the same autoencoder architecture, i.e. one hidden layer of dimension $5d$ in both the encoder and the decoder parts of the network. This choice is somewhat arbitrary and was determined empirically without a thorough optimization.

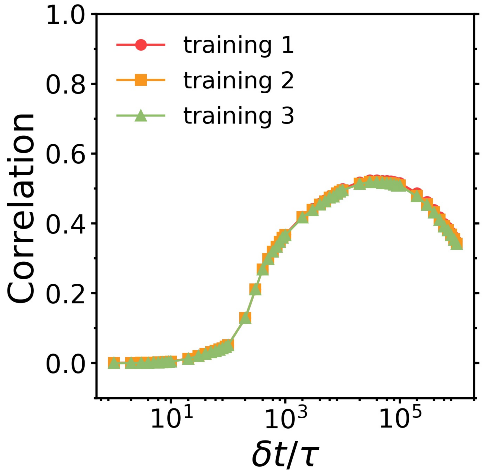

Supplementary Figure 3: Spearman's rank correlation between the particles' dynamic propensity D_i and their membership probability $P_{\rm red}(i)$ obtained with three different trainings of the UML method for the large particles of the Wahnström system.

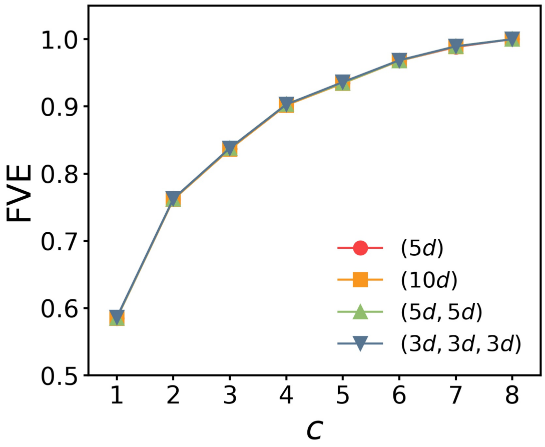

Supplementary Figure 4: FVE as a function of c obtained with different autoencoder architectures for the large particles of the Wahnström system.

In this section, we compare the results obtained with different autoencoder architectures with either a larger hidden layer, or multiple hidden layers. We specify the number and the dimensions of the hidden layers with a tuple of the form $(h_1, h_2, \dots)$, whose dimension correspond to the number of hidden layers, and whose components indicate the specific size of the layers.

Supplementary Figure 4 shows the FVE as a function of c obtained with different architectures for the large particles of the Wahnström system at density $\rho^* = 0.81$ and temperature $T^* = 0.7$. The results are approximately equal for all the architectures considered, indicating that there is no advantage in using a deeper autoencoder.

C. Bottleneck dimensionality

As explained in the Supplementary Methods, we choose the optimal dimensionality of the bottleneck, c, as the smallest value for which the FVE is at least 0.75. This choice guarantees that at least 75% of the variance of the original data can be explained by the input reconstruction, meaning that only a small part of the original information is discarded by reducing the dimensionality. However, 75% is an arbitrary choice, and it is not obviously clear how the final results might depend on this choice.

To answer this question, we performed a new analysis of the three main snapshots presented in the paper with a different choice of the bottleneck size, c. For each value of c considered, we trained a new autoencoder and subsequently performed a new clustering. A comparison

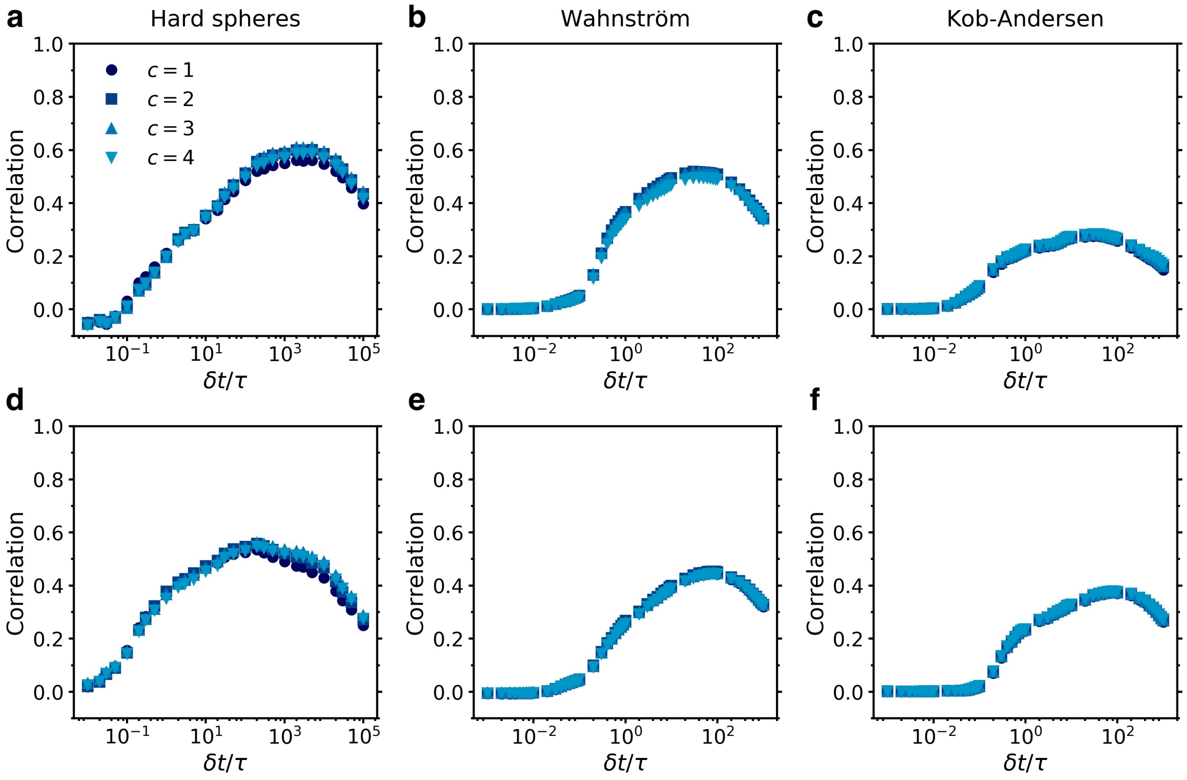

Supplementary Figure 5: Spearman's rank correlation between the particles' dynamic propensity D_i and their membership probability $P_{\mathrm{red}}(i)$ obtained with a different dimensionality of the bottleneck, c, for **a-c** large and **d-f** small particles in the hard sphere (**a**, **d**), Wahnström (**b**, **e**), and Kob-Andersen (**c**, **f**) systems at the highest degree of supercooling.

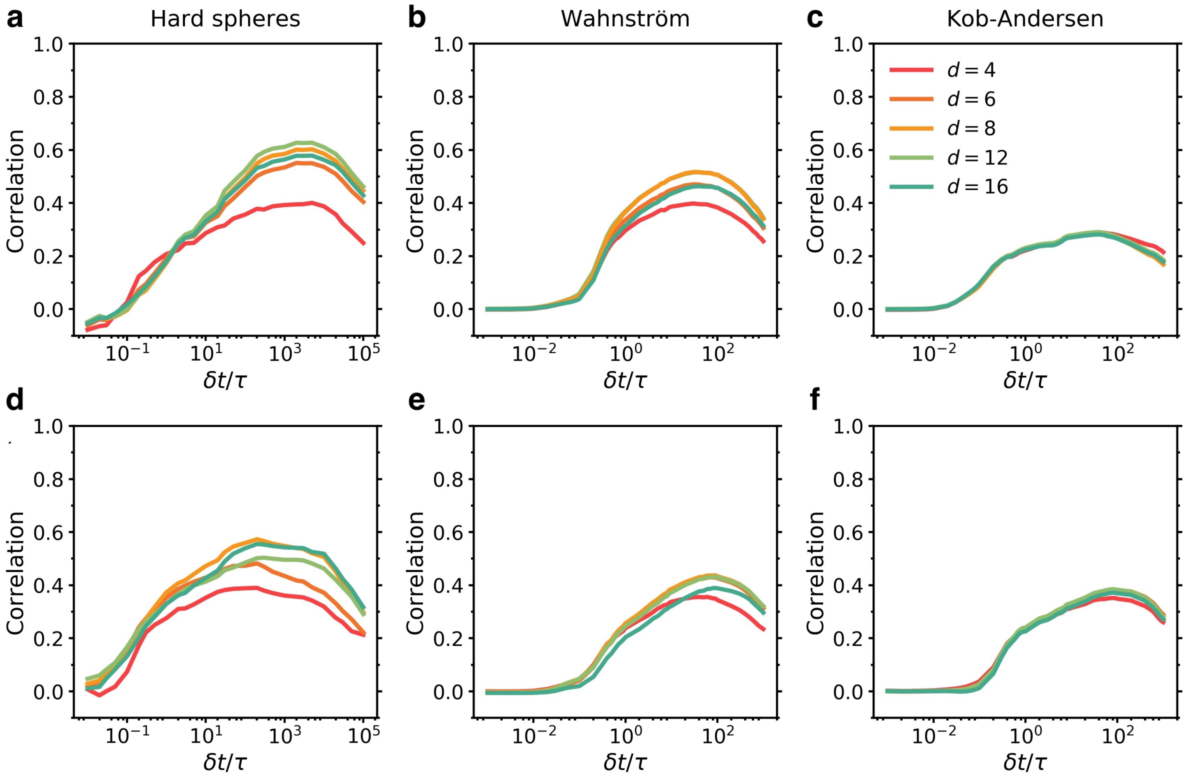

Supplementary Figure 6: Spearman's rank correlation between the particles' dynamic propensity D_i and their membership probability $P_{\mathrm{red}}(i)$ obtained with a different dimensionality of the input vectors, d, for **a-c** large and **d-f** small particles in the hard sphere (**a**, **d**), Wahnström (**b**, **e**), and Kob-Andersen (**c**, **f**) systems at the highest degree of supercooling.

of the final results obtained with $c = 1, 2, 3, 4$ is shown in Supplementary Figure 5. In all cases the results are almost unaffected by changes in the chosen value of c.

D. Input dimensionality

As explained in the methods section of the main text, our description of the local environment is encoded into a vector of $d = 8$ BOPs, $\bar{q}_l$ with $l = 1, 2, \ldots, d$, which is then used as the input of the UML method. One could in principle consider a larger or smaller set of BOPs with the obvious consequences of having a more or less complete description of a particle's local environment. In general, using a smaller set of BOPs has the risk that one might miss the relevant structural differences in the system, while including more BOPs should give a better description of the structure and, hence, a better classification. In our previous study [2], where we applied the same UML approach to identify different crystal phases in colloidal systems, we found a choice of $d = 8$ to be sufficiently large for all the systems examined. In this study, however, it is not obviously clear how the results, in particular the correlations with the dynamics, would be affected by a different choice of d.

To explore this, we repeated the analysis of the three main snapshots presented in the paper with different choices of d: $d = 4, 6, 8, 12, 16$. For each value considered, we trained a new autoencoder and subsequently performed a new clustering. Additionally, in every case we determined the optimal dimensionality of the bottleneck, c, as the smallest value with a FVE of at least 0.75. Note that, with this choice, c increases with d. The results for the large and small particles of the three systems are shown in Supplementary Figure 6.

For all systems we find a choice of $d = 8$ to be nearly optimal for both species of particles. Specifically, for the hard sphere and Wahnström systems, the correlations get worse using $d < 8$, clearly indicating that at least part of the relevant information that correlates with the dynamics is encoded in BOPs with higher l. Additionally, increasing d leads to essentially the same or even worse correlations than $d = 8$, indicating that the BOPs with $l > 8$ capture structural differences that poorly correlate with the dynamics. For the Kob-Andersen system, instead, the correlations are only weekly affected by a different choice of d.

SUPPLEMENTARY NOTE 2: COMPARISON TO SUPERVISED METHODS FOR LINKING STRUCTURE AND DYNAMICS

As shown in the main text, we find significant correlations between the structural heterogeneities found by the UML order parameter $P_{\rm red}$ and the dynamical heterogeneities encoded in the dynamic propensity. In order to obtain an estimate of how much dynamical information is retained during the dimensionality reduction, it is interesting to compare these correlations to the results of supervised machine learning techniques. These supervised machine learning techniques explicitly fit dynamical data based on a input set of structural features.

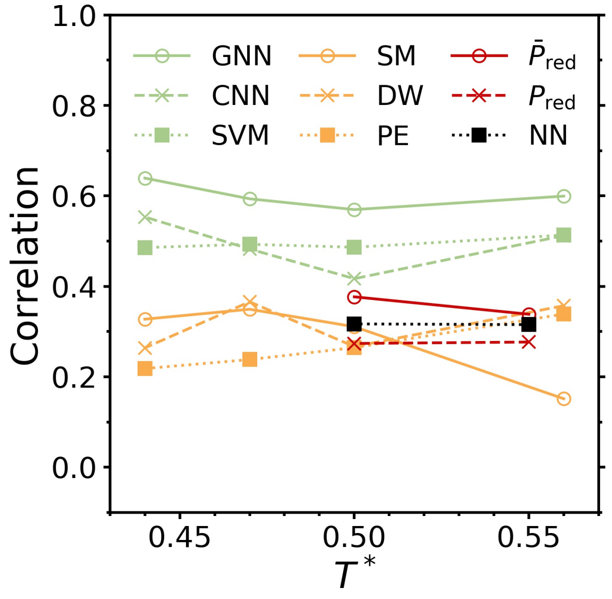

Supplementary Figure 7: Comparison of the Pearson's correlation coefficient between different order parameters and the dynamic propensity. We compare our results on the Kob-Andersen mixture to previous results taken from Ref. [13]. The methods explored there include three supervised machine learning methods: graph neural networks (GNN), convolutional neural networks (CNN), and support-vector machines (SVM) [14], which are all trained explicitly to fit dynamic propensity data. In addition, Ref. [13] examined three physics-based order parameters, based on soft modes [15], the Debye-Waller factor [16], and the potential energy [17, 18]. We compare these results to the prediction given by our UML order parameter $P_{\rm red}$, the locally averaged $\bar{P}_{\rm red}$, and a simple neural network (NN) taking the same vector $\mathbf{Q}$ as input as our autoencoder and fitted to the dynamic propensity.

For the Kob-Andersen mixture, Ref. 13 provides an excellent set of benchmarking data for various temperatures. Specifically, they compare the performance of state-of-the-art supervised ML techniques for predicting dynamic propensity based on structure. This includes the support-vector machine (SVM) approach introduced in Ref. [14], as well as more advanced machine learning methods: convolutional neural networks (CNN) and graph neural networks (GNN). As shown in Supplementary Figure 7, these algorithms all provide better predictions of the dynamic propensity than our $P_{\rm red}$ and its locally averaged version $\bar{P}_{\rm red}$. This is expected: explicitly drawing on dynamical data should always allow for a better fit than relying purely on structural information. It is however, interesting to note that $\bar{P}_{\rm red}$ does outperform all three of the physical order parameters used in

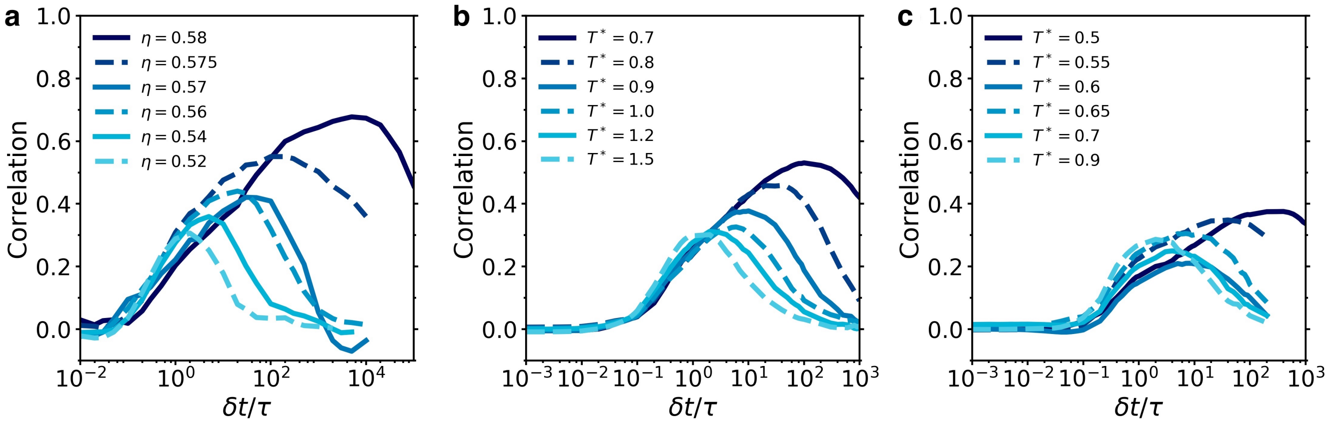

Supplementary Figure 8: Correlation between the locally averaged $\bar{P}_{\rm red}$ and dynamic propensity for small particles for **(a)** hard spheres, **(b)** Wahnström and **(c)** Kob-Andersen. Note that the averaging radius for $\bar{P}_{\rm red}$ is $2\sigma_A$ in all cases.

Ref. [13] as benchmarks (also plotted in Supplementary Figure 7), which are based on soft modes in the system [15], the local Debye-Waller factor [16], and the potential energy [17, 18].

One key factor in the ability of a (supervised or unsupervised) machine-learned order parameter to predict dynamic propensity is its input data. For our UML parameter, we use a relatively small set of eight bond-order parameters, and it is interesting to see how well a supervised learning algorithm performs with such an input. To test this, we performed a supervised machine learning analysis on our state points included in Supplementary Figure 7. Specifically, we trained a standard neural network (3 hidden layers, with a width of $2d$ each), to fit the dynamic propensity of each particle in the snapshot based on the input vector $\mathbf{Q}$. The result is shown as the black dashed line in Supplementary Figure 7. From this, we can make two observations. First, the correlations for $P_{\rm red}$ are only slightly lower than those for the NN, indicating that the UML approach retains the vast majority of the dynamical data encoded in the full set of bond order parameters. Second, for Kob-Andersen, the small set of bond order parameters is not a great predictor for dynamic propensity: the NN scores significantly worse than e.g. the SVM approach, which used 440 hand-crafted structural features as input [13, 19]. Based on this, it is possible that a larger set of input data per particle could also improve our UML approach, although we leave this for future work.

SUPPLEMENTARY NOTE 3: DYNAMIC PROPENSITY CORRELATIONS FOR SMALL PARTICLES

In the main text, we report the correlation between $P_{\rm red}$ and dynamic propensity for the large particles in all three of our glass forming models at different degrees of supercooling. For completeness, in Supplementary Figure 8 we show the same data for the small particles, where we observe approximately the same results.

SUPPLEMENTARY NOTE 4: LOCAL AVERAGING OF THE STRUCTURAL ORDER PARAMETER

In the main text, we locally average the structural order parameter found by the UML method, $P_{\rm red}$, over a spherical local region of radius $r_c = 2\sigma_A$ for all systems. Note that, as our method is purely based on structural information, this radius was not optimized in order to maximize the correlations with the dynamics.

In this section we explore how the choice of such a radius influences the correlations with the dynamics, and whether we can extract a growing length scale with the degree of supercooling of the system.

A. Correlations for different cutoff radii

Here, we consider the three main snapshots analyzed in the main text. For each snapshot, we compute the locally averaged $\bar{P}_{\rm red}$ using three different cutoff radii, $r_c/\sigma_A = 1, 2, 3$, and see how the Spearman correlation with the dynamic propensity is influenced by this choice. A comparison of the correlations obtained for the three systems is shown in Supplementary Figure 9.

In general, we find that the maximum in the correlation shifts to longer times by increasing r_c. Moreover, in all cases, the correlation improves going from $r_c/\sigma_A = 1$ to $r_c/\sigma_A = 2$. In particular, for both species of particles in the hard sphere system and for the large particles of the Wahnström system the correlation gets worse for $r_c/\sigma_A = 3$. In the remaining cases (small particles of the Wahnström system and both species of particle in the Kob-Andersen system), we find very small differences between $r_c/\sigma_A = 2$ and $r_c/\sigma_A = 3$.

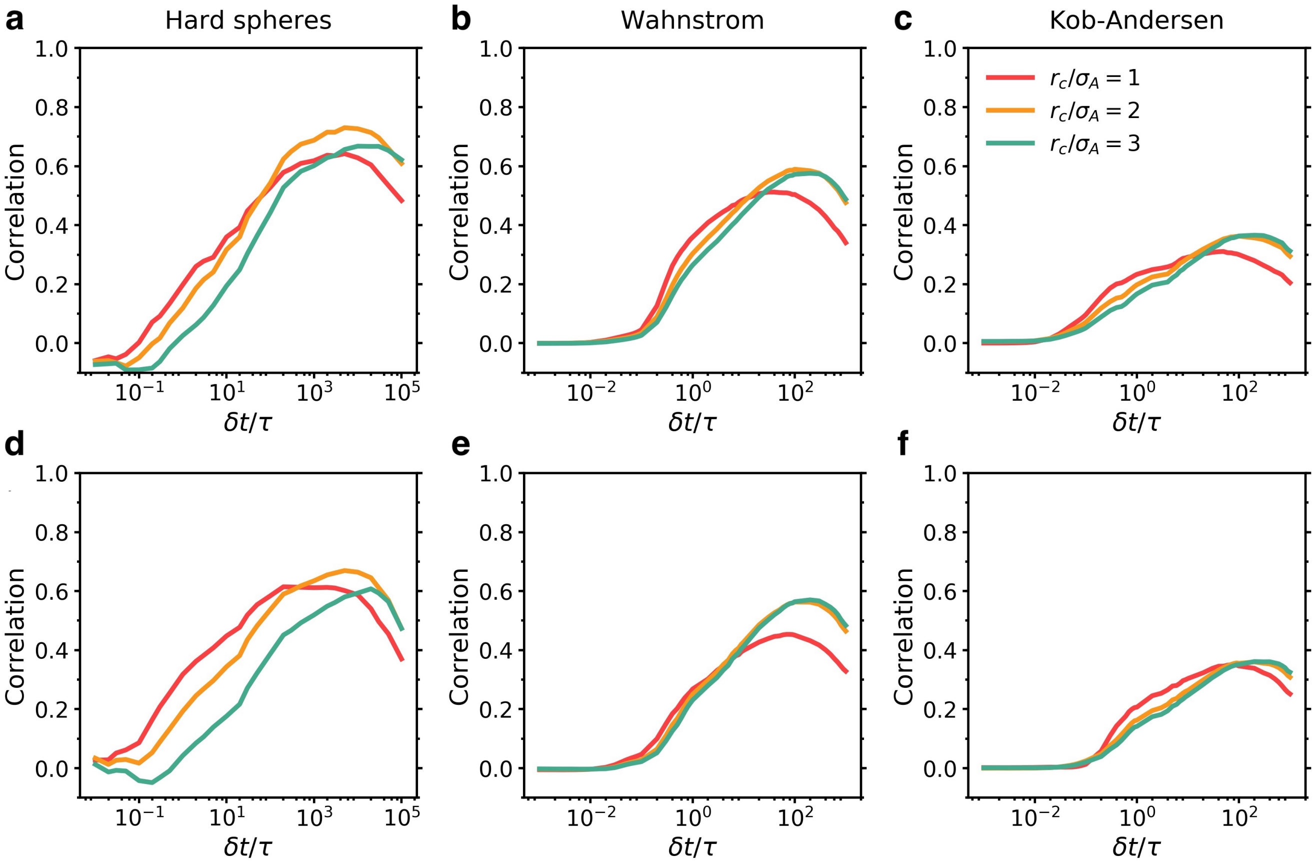

Supplementary Figure 9: Spearman's rank correlation between the particles' dynamic propensity D_i and their locally averaged membership probability $\bar{P}_{\rm red}(i)$ obtained with a different averaging cutoffs, r_c, for **a-c** large and **d-f** small particles in the hard sphere (**a**, **d**), Wahnström (**b**, **e**), and Kob-Andersen (**c**, **f**) systems at the highest degree of supercooling.

B. Length scale

In order to explore whether we can extract a growing length scale with the supercooling of the system, we now consider the three systems at different degrees of supercooling, i.e. different packing fraction η for hard spheres, and different temperature T^* for the Wahnström and Kob-Andersen systems.

For each system, we optimize the cutoff radius by maximizing the correlations of $\bar{P}_{\rm red}$ with the dynamic propensity. Specifically, we compute $\bar{P}_{\rm red}$ using different radii ($r_c/\sigma_A = 1.0, 1.5, 2.0, 2.5, 3.0, 3.5, 4.0$), and choose the optimal radius r_c^* as the one for which the maximum in the correlation between $\bar{P}_{\rm red}$ and the dynamic propensity is the highest. Supplementary Figure 10 shows the results of this analysis for the three systems.

For the Wahnström and especially for the Kob-Andersen systems we clearly find that the optimal cutoff increases with the degree of supercooling. This behaviour is less pronounced and more irregular for the hard-sphere systems, which might be caused by the relatively small system size considered for such systems (only $N = 2000$ particles compared to the $N = 64000$ particles of the Wahnström and Kob-Andersen systems). Nonetheless, the largest optimal cutoff is always found at the deepest supercooling.

It should be noted that this length scale is not a purely structural one as we find the optimal r_c^* by comparing to dynamical data. As a result, it seems likely that the optimal r_c^* is linked to the dynamical correlation length of the system rather than being indicative of a growing static length scale.

SUPPLEMENTARY NOTE 5: CORRELATIONS IN THE TRAINED ORDER PARAMETERS

The next question we want to address is whether our trained order parameter shows signs of a growing *structural* correlation length in our systems. To investigate this, we calculate the radial autocorrelation function associated with $P_{\rm red}$, defined as:

$$C_{\rm red}(r) = \frac{\sum_{i \neq j}^{N} p_i p_j \delta(\mathbf{r} + \mathbf{r}_i - \mathbf{r}_j)}{\sum_{i \neq j}^{N} \delta(\mathbf{r} + \mathbf{r}_i - \mathbf{r}_j)}, \quad (3)$$

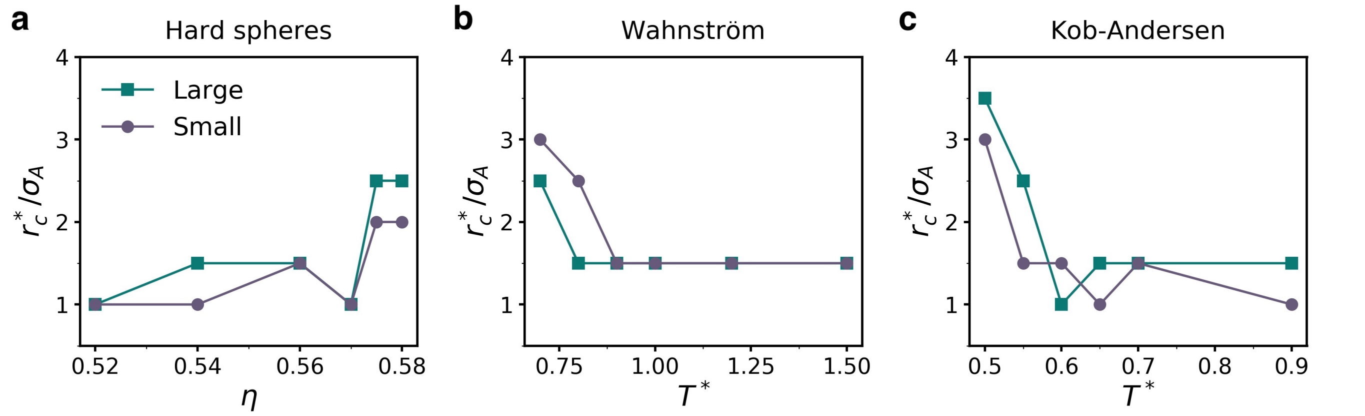

Supplementary Figure 10: Cutoff distance r_c^* that maximises the correlations with the dynamic propensity as a function of the degree of supercooling for the **a** hard sphere, **b** Wahnström, and **c** Kob-Andersen systems.

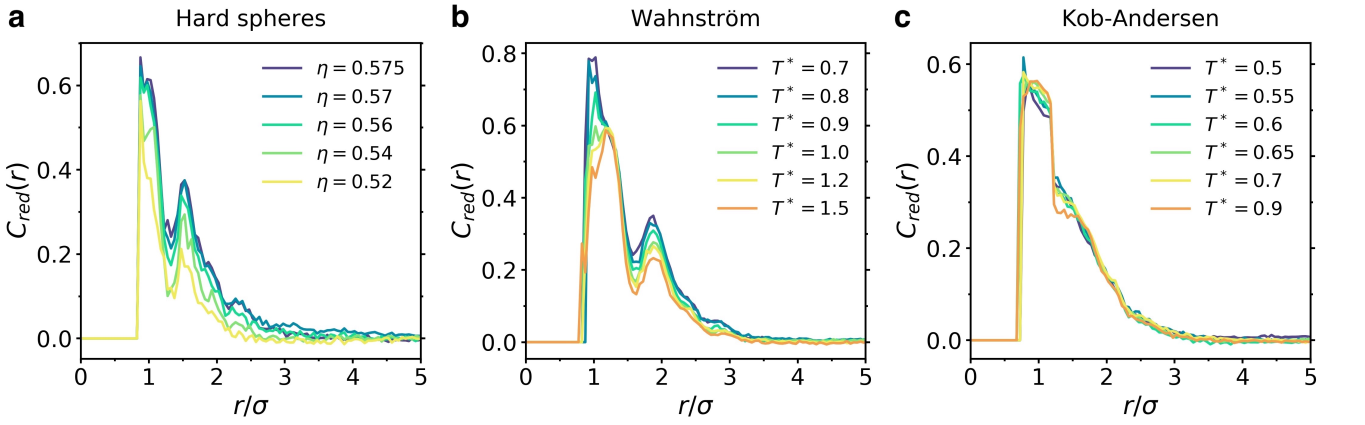

Supplementary Figure 11: Radial auto-correlation function $C_{\rm red}$ associated with the trained order parameter at different degrees of supercooling for **a** hard sphere, **b** Wahnström, and **c** Kob-Andersen systems.

where $\mathbf{r}_i$ is the position of particle i, and the normalized order parameter p_i of each particle is defined as

$$p_i = \frac{P_{\rm red}(i) - \langle P_{\rm red}\rangle}{\sqrt{\langle P_{\rm red}\rangle^2 - \langle P_{\rm red}\rangle^2}}, \tag{4}$$

with $\langle P_{\rm red}\rangle$ the average value of $P_{\rm red}$ taken over all particles.

The resulting correlation functions for all three systems are shown in Supplementary Figure 11. While we do observe some increase in overall correlations as each system is supercooled, the range over which these correlations exist does not grow appreciably. This is in contrast to the growing dynamical length scale associated with r_c^* in Supplementary Figure 10.

SUPPLEMENTARY NOTE 6: BOND ORDER PARAMETERS IN THE TWO CLUSTERS OF SMALL PARTICLES

In the main text, we report the mean value of all bond order parameters $q_1, \cdots, q_8$ for both red (more mobile, $P_{\rm red} > 0.5$) and white clusters ($P_{\rm red} < 0.5$) of large particles in all three of our glass forming models. For completeness, in Supplementary Figure 12 we show the same data for the small particles, where we observe approximately the same results.

SUPPLEMENTARY NOTE 7: MODE COUPLING THEORY FITS

For each of our model glass formers, we extract the mode coupling theory (MCT) critical temperature (T_{MCT}) or packing fraction (η_{MCT}) by performing standard MCT fits to the scaling of the structural relaxation

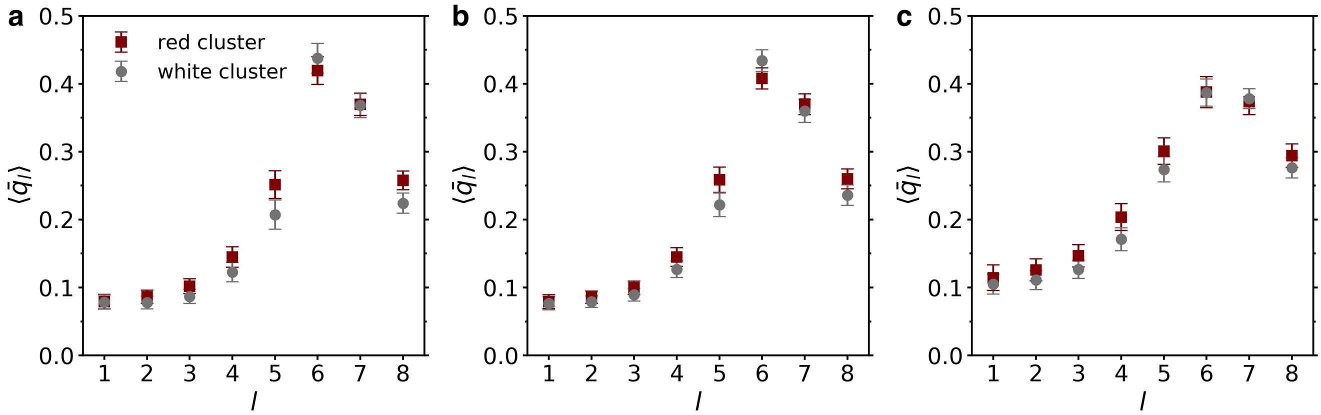

Supplementary Figure 12: Mean values of the BOPs for the small particles in the two clusters identified by the unsupervised learning in **a** hard spheres, **b** Wahnström, and **c** Kob-Andersen systems. Bars are the standard deviations.

	γ	T^*_{MCT}, η_{MCT}
WS	2.07 ± 0.09	0.652 ± 0.004
KA	2.5 ± 0.3	0.42 ± 0.01
HS	3.0^*	0.5837 ± 0.0007

Supplementary Table I: MCT fit parameters (Supplementary Equation 5). For the exponent γ of the HS case, we followed Ref. 20 and used a fixed value.

time τ_α as a function of temperature (or packing fraction):

$$\tau_\alpha \propto \frac{1}{\varepsilon^\gamma} \quad (5)$$

where ε is the distance from the MCT critical point, i.e. $\varepsilon = T - T_{MCT}$ for Kob-Andersen and Wahnström and $\varepsilon = \eta_{MCT} - \eta$ for binary hard spheres. The fits are displayed in Supplementary Figure 13 and the resulting fit parameters are presented in Supplementary Table I.

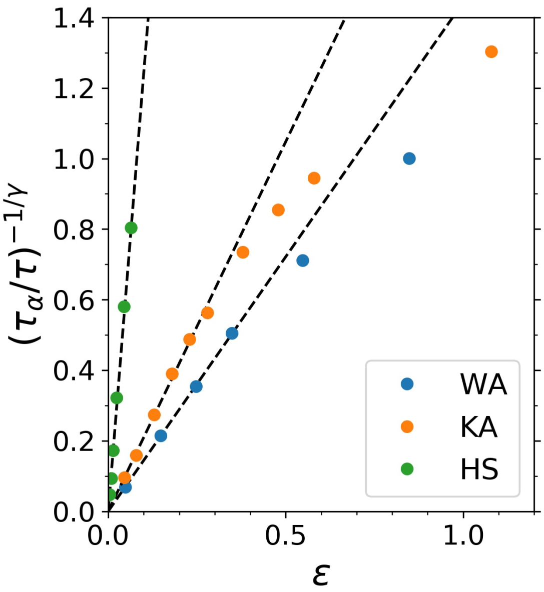

Supplementary Figure 13: Fits used to extract the mode coupling temperatures and packing fraction in Table I. The points are measurements of the structural relaxation time τ_α from our simulations, and the dashed lines are fits following Eq. 5.

SUPPLEMENTARY NOTE 8: SANN VS. CUTOFF IN KOB-ANDERSEN

In order to describe the local environment of each particle i in terms of BOPs, needed for $\mathbf{Q}(i)$, we require a definition of what neighbors we include in the bond order calculation. For both hard spheres and Wahnström, we used the definition of nearest neighbour from the solid angle nearest neighbor (SANN) algorithm [21]. This algorithm is known to work well for hard spheres, and as Wahnström is very simliar, we expected it to work well here as well. For Kob-Andersen, we also initially used SANN, but later found that using a tuned fixed cutoff radius worked better. In the following we compare the results of SANN and our cutoff of 1.2 σ_A. In particular, we analyse a snapshot of a glassy Kob-Andersen mixture at density $\rho^* = 1.2$ and temperature $T^* = 0.5$.

Supplementary Figure 14 shows a comparison of the Spearman's rank correlation between the particles' dynamic propensity D_i and their membership probability $P_{\rm red}(i)$ obtained by using either SANN or a fixed a cutoff radius.

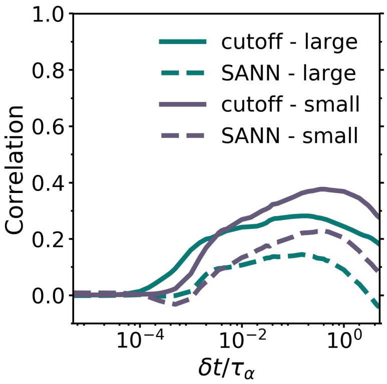

Supplementary Figure 14: Comparison of the Spearman's rank correlation between the particles' dynamic propensity D_i and their membership probability $P_{\text{red}}(i)$ obtained by using either SANN or a cutoff radius of $r_c = 1.2\sigma_A$ for both large and small particles.

SUPPLEMENTARY NOTE 9: PEARSON VS. SPEARMAN CORRELATION

All our results are presented in terms of the Spearman's rank correlation between the structural order parameter found by the UML method and the dynamic propensities of the particles. The Spearman's rank correlation is a measure of how strongly the relationship between two variables can be described by a monotonic function (whether linear or not).

However, most of the other works, where supervised machine learning is used to predict the dynamics of particles in glassy systems, use instead the Pearson correlation as a measure of the performance of their methods, which complicates a direct comparison.

Note that our UML method develops an order parameter that is purely based on local structure and does not rely on any dynamical information. As such, there is no obvious reason to believe that P_{red} and the dynamic propensity should be linearly related, which is why we decided to use the Spearman's rank correlation. Nonetheless, we find that two types of correlations are very similar for all systems analyzed. As an example, Supplementary Figure 15 shows a comparison of the Pearson and Spearman's rank correlations between $\bar{P}_{\text{red}}$ (averaged over a local neighborhood of radius $r_c/\sigma_A = 2$) and the dynamic propensity for the large particles of the three main snapshots analyzed in the paper.

SUPPLEMENTARY REFERENCES

[1] Pedregosa, F. *et al.* Scikit-learn: Machine learning in Python. *J. Mach. Learn. Res.* **12**, 2825 (2011).

[2] Boattini, E., Dijkstra, M. & Filion, L. Unsupervised learning for local structure detection in colloidal systems. *J. Chem. Phys.* **151**, 154901 (2019).

[3] Rumelhart, D. E., Hinton, G. E. & Williams, R. J. Parallel distributed processing: Explorations in the microstructure of cognition, Vol. 1. chap. Learning Internal Representations by Error Propagation, 318–362 (MIT Press, Cambridge, MA, USA, 1986).

[4] Kramer, M. A. Nonlinear principal component analysis using autoassociative neural networks. *AIChE J.* **37**, 233–243 (1991).

[5] Scholz, M. & Vigário, R. Nonlinear PCA: a new hierarchical approach. In *ESANN* (2002).

[6] Goodfellow, I., Bengio, Y. & Courville, A. *Deep Learning* (The MIT Press, 2016).

[7] Bishop, C. M. *Neural Networks for Pattern Recognition* (Oxford University Press, Inc., New York, NY, USA, 1995).

[8] Glorot, X. & Bengio, Y. Understanding the difficulty of training deep feedforward neural networks. In *Proceedings of the thirteenth international conference on artificial intelligence and statistics*, 249–256 (2010).

[9] Rumelhart, D. E., Hinton, G. E. & Williams, R. J. Learning representations by back-propagating errors. *Nature* **323**, 533 (1986).

[10] Sutskever, I., Martens, J., Dahl, G. & Hinton, G. On the importance of initialization and momentum in deep learning. In *Proceedings of the 30th International Conference on Machine Learning*, 1139–1147 (JMLR, 2013). URL `http://dl.acm.org/citation.cfm?id=3042817.3043064`.

[11] Dempster, A., Laird, N. & Rubin, D. Maximum likelihood from incomplete data via the EM algorithm. *J. R. Stat. Soc. Series B* **39**, 1 (1977).

[12] Schwarz, G. Estimating the dimension of a model. *Ann. Statist.* **6**, 461 (1978).

[13] Bapst, V. *et al.* Unveiling the predictive power of static structure in glassy systems. *Nature Physics* **16**, 448–454 (2020).

[14] Cubuk, E. D. *et al.* Identifying structural flow defects in disordered solids using machine-learning methods. *Phys. Rev. Lett.* **114**, 108001 (2015).

[15] Widmer-Cooper, A., Perry, H., Harrowell, P. & Reichman, D. R. Irreversible reorganization in a supercooled liquid originates from localized soft modes. *Nat. Phys.* **4**, 711–715 (2008).

[16] Widmer-Cooper, A. & Harrowell, P. Predicting the long-time dynamic heterogeneity in a supercooled liquid on the basis of short-time heterogeneities. *Phys. Rev. Lett.* **96**, 185701 (2006).

[17] Berthier, L. & Jack, R. L. Structure and dynamics of glass formers: Predictability at large length scales. *Phys. Rev. E* **76**, 041509 (2007).

[18] Doliwa, B. & Heuer, A. What does the potential energy landscape tell us about the dynamics of supercooled liquids and glasses? *Phys. Rev. Lett.* **91**, 235501 (2003).

[19] Schoenholz, S. S., Cubuk, E. D., Sussman, D. M., Kaxiras, E. & Liu, A. J. A structural approach to relaxation in glassy liquids. *Nat. Phys.* **12**, 469–471 (2016).

[20] Foffi, G., Götze, W., Sciortino, F., Tartaglia, P. & Voigtmann, T. α-relaxation processes in binary hard-sphere mixtures. *Phys. Rev. E* **69**, 011505 (2004).

[21] Van Meel, J. A., Filion, L., Valeriani, C. & Frenkel, D. A parameter-free, solid-angle based, nearest-neighbor algorithm. *J. Chem. Phys.* **136**, 234107 (2012).

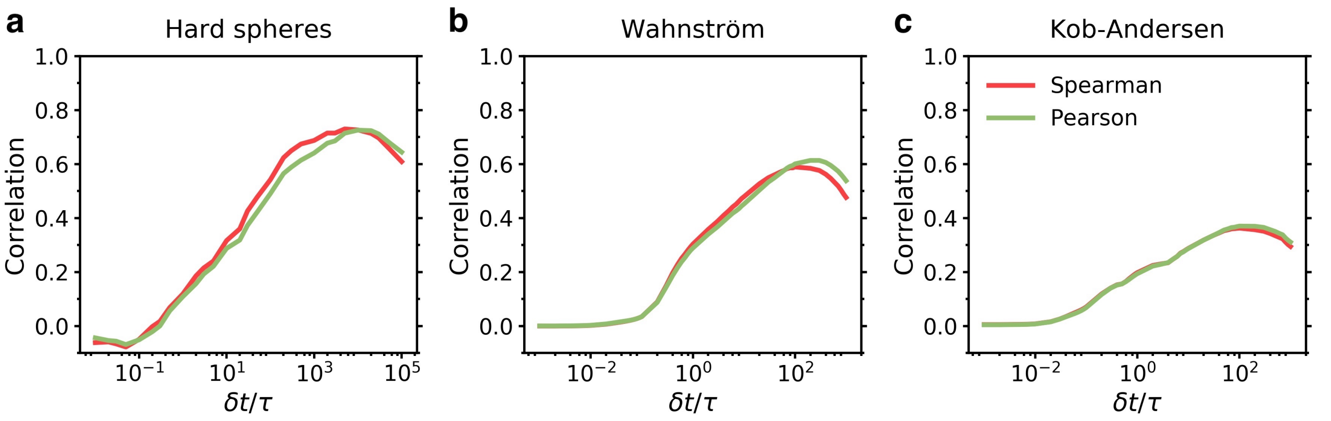

Supplementary Figure 15: Comparison of the Pearson and Spearman's rank correlations between $\bar{P}_{\mathrm{red}}$ and the dynamic propensity for the large particles of the **a** hard sphere, **b** Wahnström, and **c** Kob-Andersen systems.
